# Supplementary material for: Supercluster-coupled crystal growth in metallic glass forming liquids
Source: Nat Commun. 2019 Feb 22;10:915. doi: 10.1038/s41467-019-08898-4 (PMC6385493; doi:10.1038/s41467-019-08898-4)
Supplement: Supplementary file 1 — Supplementary Information [file 41467_2019_8898_MOESM1_ESM.pdf]

Supplementary Information for

Supercluster-Coupled Crystal Growth in Metallic Glass Forming Liquids

Yujun Xie*, Sungwoo Sohn*, Minglei Wang, Huolin Xin, Yeonwoong Jung, Mark D. Shattuck, Corey S. O'Hern, Jan Schroers, Judy J. Cha

* These authors contributed equally.

Supplementary Note 1: Quantification of a crystal growth front using intensity profiles of Fourier filtered atomic resolution TEM images

Supplementary Fig. 2 shows a step-by-step procedure for obtaining a Fourier filtered TEM image, which was used to determine the growth front position and growth rate. Supplementary Fig. 2a shows a TEM snapshot from the isothermal crystallization movie of a 23 nm nanorod at 360°C (Supplementary Movie 3). The TEM image was obtained by summing 200 frames of the *in situ* movie to increase the signal-to-noise ratio of the image. Since the movies were taken at 400 frames per second, summing 200 frames provides a temporal resolution of 0.5 sec. The area marked by the red dashed line in Supplementary Fig. 2a contains the growth front that we track. The fast Fourier transform (FFT) diffractogram of the area was obtained using Digital Micrograph (version 3.01 from Gatan Inc.), as shown in Supplementary Fig. 2c. The bright spots in the FFT pattern indicates the presence of a crystalline phase. We apply an annular mask to the FFT pattern, in which the inner radius of the mask excludes the center spot while the outer radius of the mask covers most of the main spots (Supplementary Fig. 2d). We constructed a Fourier filtered image by inverse fast Fourier transforming the data in Supplementary Fig. 2d, as shown in Supplementary Fig. 2e. To quantify the growth front, an intensity profile from the dashed area in cyan was generated along the direction perpendicular to the (200) crystallographic plane (Supplementary Fig. 2f). The position of the growth front was determined as the location at which the intensity envelope was below 10 % of the maximum.

Supplementary Note 2: Determination of the growth rate during isothermal crystallization experiments

The crystal growth rate is strongly affected by the crystallographic orientation of a grain. We first tracked the grain growth in two orientations. For example, for isothermal crystallization of a 65 nm nanorod from the liquid melt state, the average growth rates were measured to be ~ 0.3 nm s^{-1} and ~ 6 nm s^{-1} along d_{200} and d_{110} directions respectively. The growth rate along the densely packed direction is ~ 20 times faster than the growth rate perpendicular to the largest inter-planar spacing plane. For the growth rate comparison as a function of the nanorod diameter and thermal history (Fig. 3c and 3d of the main text), we only use the growth rate perpendicular to the (200) plane for the following reasons: i) a larger inter-planar spacing generates a larger change in contrast, making quantitative analysis more accurate, and ii) a slower growth rate allows us to average more frames for a better signal-to-noise ratio while ensuring that the time resolution after averaging is still high enough to track the crystallization process.

Supplementary Note 3: Details of the molecular dynamics simulations of crystallization in binary Lennard-Jones metallic glasses

Thermal quenching protocol for preparing glasses

We investigated isothermal growth kinetics of a crystal invading an amorphous sample using molecular dynamics (MD) simulations of binary Lennard-Jones atoms. We first initialized the sample in a FCC crystal structure. We then fixed the positions of the atoms in the central region of the sample (~ 5 layers) and equilibrated the system at high temperature above the melting temperature $T_0 > 2.6$ (in units of ε/k_b, where ε is the depth of the Lennard-Jones potential and k_b is

Boltzmann's constant). In Supplementary Fig. 3, we show that the total potential energy per atom, *U/N,* increases rapidly near $T \approx 2.6$, which indicates the melting transition. We then cooled the system using a linear ramp, $T = T_0 - R \cdot t$, with cooling rate, R, to a final temperature $T = 0.01$, much below the glass transition temperature $T_g \approx 0.38$ [1,2]. We studied cooling rates higher than the critical cooling rate so that edges of the sample were initially amorphous at $T = 0.01$ with the fixed FCC crystal in the central region. The critical cooling rate was determined to be ~ 0.02 (in units of $\varepsilon/(\sigma k_b) \sqrt{\frac{\varepsilon}{m}}$, where σ is the diameter and m is the mass of the smaller atom) at which the probability of crystallization is 0.5 for the system (Supplementary Fig. 4). After the quench to $T = 0.01$, we heat the system in the fast heating rate limit to $T = 0.1$ and run the simulations at fixed temperature for times $t = 100$ (in units of $\sqrt{\frac{m\sigma^2}{\varepsilon}}$), which is comparable to the structural relaxation time near T_g. During the isothermal simulations at $T = 0.1$, the crystal region in the center of the sample expands into the amorphous regions.

Crystal growth rate measurements in MD simulations

We now describe our method to quantify the crystallization kinetics during the isothermal simulations at temperature $T = 0.1$. We identify crystal-like atoms that reside in structurally ordered local environments. We detect FCC crystalline order using the Voronoi surface area-weighted bond orientational order parameter for each particle, as shown in equation (1) [3]:

$$q_{6m}(i) = \frac{\sum_{j=1}^{N_b} A_{ij} Y_{6m}(\theta(\vec{r}_{ij}), \phi(\vec{r}_{ij}))}{\sum_{j=1}^{N_b} A_{ij}} \qquad (1),$$

where $Y_{6m}(\theta(\vec{r}_{ij}), \phi(\vec{r}_{ij}))$ is the spherical harmonic of degree 6 and order $m = -6, -5, \dots, 5, 6$, $\theta(\vec{r}_{ij})$ and $\phi(\vec{r}_{ij})$ are the polar and azimuthal angles for the vector $\vec{r}_{ij}$, j is

summed over the Voronoi neighbors of particle i, N_b is the number of Voronoi neighbors, and A_{ij} is the area of the face of the Voronoi polyhedron shared by neighboring particles i and j. $q_{6m}(i)$ represents the local 6-fold bond orientational order between particle i and its Voronoi neighbors. We detect crystal-like particles by first measuring the correlation between $q_{6m}(i)$ and $q_{6m}(j)$ of neighboring particles i and j [4-6]:

$$S_{ij} = \frac{\sum_{m=-6}^{6} q_{6m}(i) q_{6m}^{*}(j)}{\left[\sum_{m=-6}^{6} |q_{6m}(i)|^2\right]^{1/2} \left[\sum_{m=-6}^{6} |q_{6m}(j)|^2\right]^{1/2}} \qquad (2).$$

For $S_{ij} > 0.7$, we consider that the bond orientational order of particles i and j is large and correlated. If particle i has more than 6 highly correlated neighbors, we define it to be a "crystal-like" particle. Otherwise, the particle is considered "liquid-like". Using this technique, for each snapshot of the system, we can determine the fraction of crystal- and liquid-like particles.

In Fig. 3e of the main text, we show the detection of crystal-like particles during simulations at constant $T = 0.1$ at two time stamps. We see that the crystalline region in the center has expanded from 5 layers of particles in the as-quenched sample to 16 layers after running the simulations at constant $T = 0.1$. We identify the leftmost and rightmost atoms in the large connected crystalline domain to determine the location of the growth front as a function of time. The presence of small crystalline seed domains that are originally separated from the crystalline region and then merge with the growing crystal gives rise to large fluctuations in the crystal growth rate.

Supplementary Note 4: Possible experimental factors that can affect crystallization kinetics other than the presence of small clusters

For the *in situ* TEM experiments, extrinsic factors, such as electron beam (e-beam) irradiation, effects of surface oxidation and carbon build-up during imaging, can significantly affect the

crystallization process. In our previous study[7], we have considered several of these experimental factors, which include composition change during experiments, electron beam (e-beam) irradiation, effects of surface oxidation layer, carbon build-up, curvature of the nanorod effect, reduced thermal conductivity for nanoscale samples and potentially poor thermal contact between the nanorods and the *in situ* TEM grid. We discussed how these factors may affect crystallization, and ruled out the possibility that they have any significant effects on the crystallization kinetics of the rods we studied. The details can be found in the Supplementary information of our previous work[7].

Supplementary Note 5: Estimation of transient times for the Pt-based MG at elevated temperature

We calculated transient times for bulk Pt-based MG based on the following equation[8]:

$$\tau_{tr} = C_{tr} \frac{{n^*}^2 {a_0}^2}{o^* D} \tag{3}$$

where C_{tr} is the adjustment factor, n^* is the number of atoms in the critical nucleus, a_0 is the average interatomic spacing, o^* is the number of surface atoms in the critical nucleus, and D is the diffusion coefficient estimated through the Stokes-Einstein relation. In the bulk sample, the transient time is estimated to be 0.128 sec at 340°C using C_{tr} = 7.9, n^* = 99, o* = 87, a_0 = 0.31 nm and D = $1.2857*10^{-16}$ m^2s^{-1} [9].

SUPPLEMENTARY FIGURES

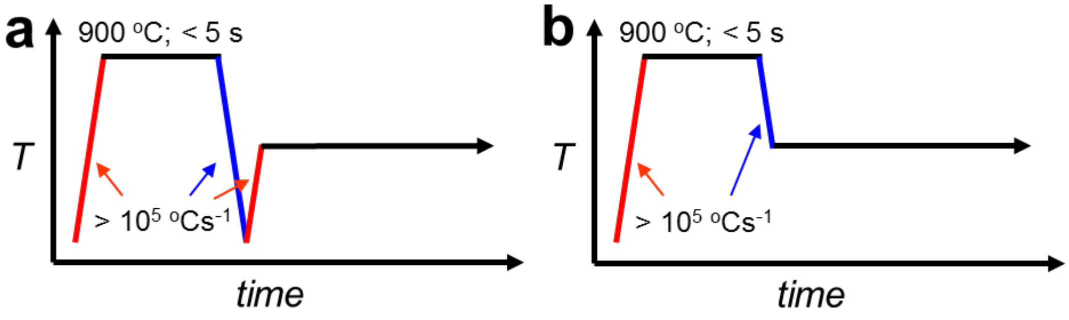

Supplementary Figure 1. Temperature-time sequences used for isothermal crystallization experiments. **a.** Isothermal crystallization annealed from the glass state. The MG rods were initially rapidly heated to 900°C for less than 5 sec, then quenched to room temperature to erase any thermal history. After the melt-quench step, the rods were brought up to the isothermal crystallization temperature rapidly and held at the temperature for the entire *in situ* experiment. Growth fronts of crystalline grains were tracked during the isothermal crystallization. **b.** Isothermal crystallization quenched from the melt state. The rods were initially rapidly heated to 900°C for less than 5 sec, then cooled to the isothermal crystallization temperature rapidly and held at the temperature for the entire *in situ* experiment. Growth fronts of crystalline grains were tracked during the isothermal crystallization.

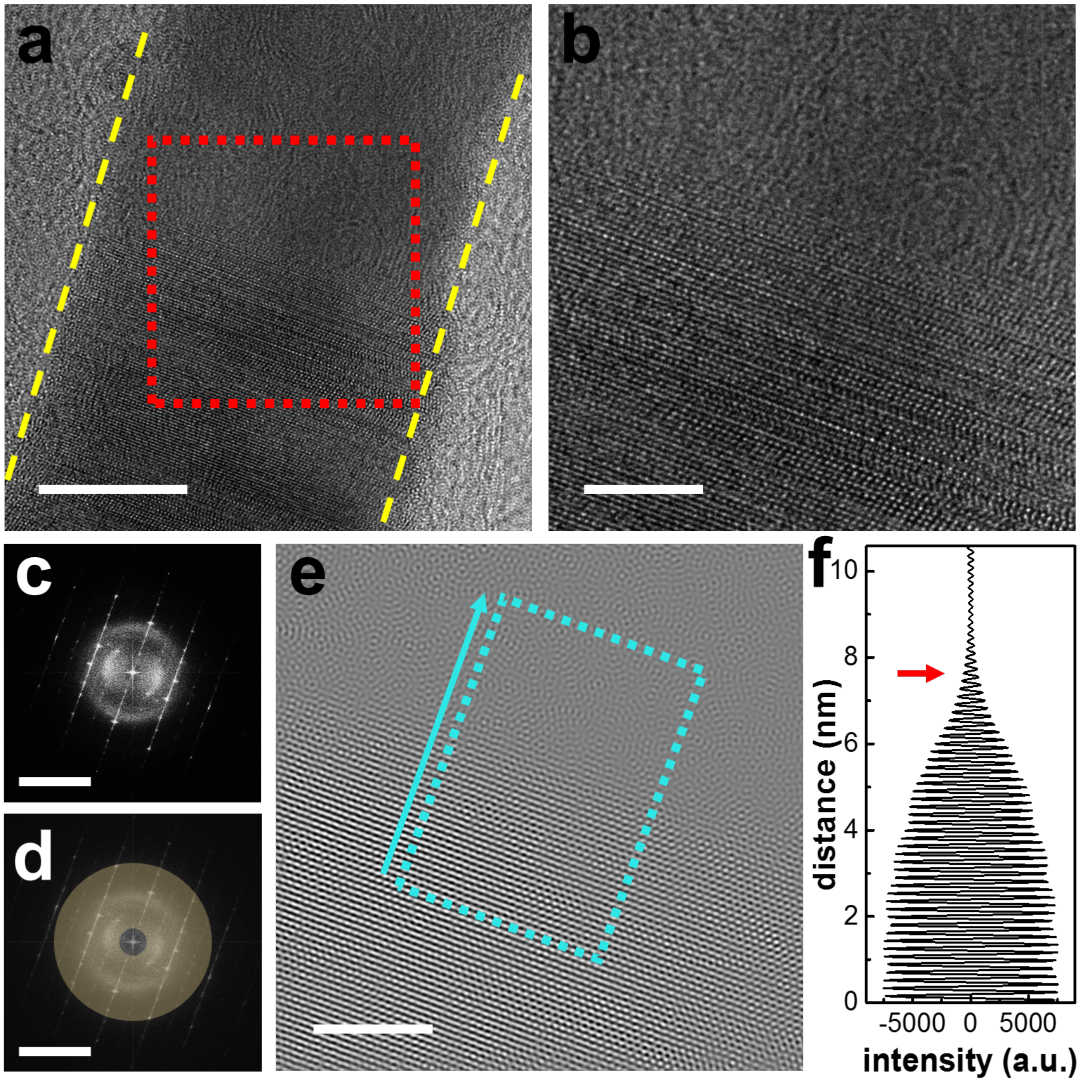

Supplementary Figure 2. Quantitative measurement of the growth front position. **a.** A high resolution snapshot obtained from the 23 nm nanorod *in situ* movie (Supplementary Movie 3). The snapshot was obtained by averaging 200 consecutive frames in the movie for increased signal-to-noise ratio. The yellow dashed line marks the edge of the MG rod. The region in red dashed line marks the region of interest (ROI). Scale bar = 10 nm. **b.** The ROI. Scale bar = 4 nm. **c.** A corresponding diffractogram of the ROI by fast Fourier transform (FFT). Scale bar = 1 nm^{-1}. **d.** An annular mask was applied to the diffractogram, removing the zero and high frequency regions. Scale bar = 1 nm^{-1}. **e.** A filtered snapshot obtained by inverse FFT of the filtered diffractogram.

The region marked by the dashed line contains the growth front. The growth direction, indicated by the arrow, is perpendicular to the (200) plane. Scale bar = 4 nm. **f.** Determination of the growth front. An intensity profile is generated from the region in the dashed box in (**e**) along the growth direction. The growth front was marked at the location at which the intensity becomes below 10 % of the maximum intensity, indicated by the arrow.

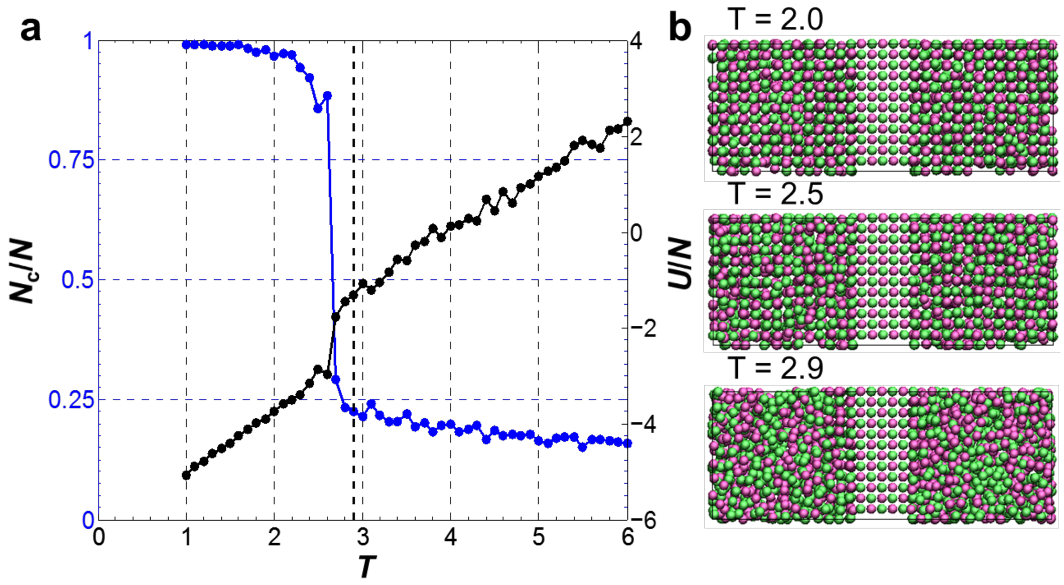

Supplementary Figure 3. a. The total potential energy per particle, *U/N*, and the fraction of crystal particles, N_c/N, are plotted as a function of reduced temperature *T* (normalized by ε/k_b) from molecular dynamics (MD) simulations of binary Lennard-Jones atoms. The system was first prepared as a face-center-cubic (FCC) crystal at low T. Particles in the center of the sample were fixed in their crystal positions and the system was heated above the melting temperature to prepare liquid states on the edges. The sudden change in both N_c/N and *U/N* near $T \approx 2.6$ indicates the onset of melting. **b.** Snapshots from the simulations at three temperatures $T = 2.0$, 2.5, and 2.9. The particles are colored pink and green to represent the A and B type atoms, respectively. When the system is heated to $T = 2.0$ and 2.5, which is below the melting temperature, the two regions on the ends that were free to relax during simulation remained largely crystalline. At $T = 2.9$ (marked as a dashed line in (**a**)), the two regions on the ends melt, while the central region remains a FCC crystal. Based on these results, for studies of crystallization upon heating, the system was prepared by first bringing it to equilibrium in the liquid state at $T = 2.9$, followed by rapid cooling to low temperature $T = 0.01$. Growth of the middle crystalline region into the glass phase was monitored by heating the system to $T = 0.1$.

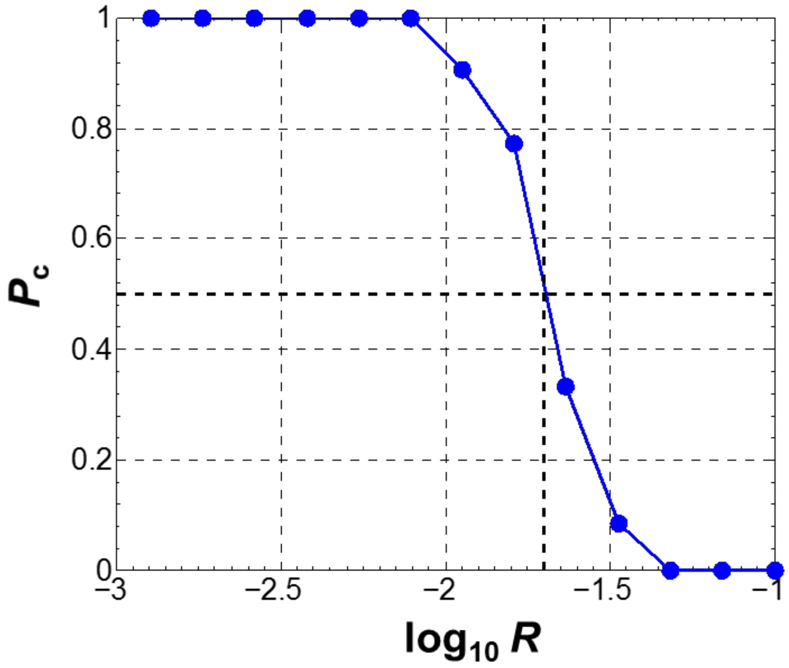

Supplementary Figure 4. Measurement of the critical cooling rate for MD simulations of binary Lennard-Jones atoms. The probability of crystallization P_c is plotted against the logarithm of the cooling rate, $\log_{10} R$, where R is in units of $\varepsilon/(\sigma k_b)\sqrt{\frac{\varepsilon}{m}}$. The probability of crystallization was obtained by running 100 simulations with different initial conditions for each cooling rate and determining which systems had crystallized. Each system was first equilibrated in the liquid state at temperature $T > 2.6$, and then cooled using a linear ramp to low temperature $T = 0.01$. We determine the number of crystal-like particles in the system as a function of time during the simulations. If at any time during the quench from the liquid to $T = 0.01$ the fraction of crystal-like particles exceeds 0.5, we consider that the sample has crystallized. $P_c \approx 1$ for cooling rates well below the critical cooling rate R_c, and $P_c \approx 0$ for $R > R_c$. We define $R_c \approx 0.02$ from $P_c(R_c) = 0.5$ indicated by the dashed lines.

Supplementary References

1 Zhang, K. *et al.* Computational studies of the glass-forming ability of model bulk metallic glasses. *The Journal of Chemical Physics* **139**, 124503 (2013).

2 Jónsson, H. & Andersen, H. C. Icosahedral Ordering in the Lennard-Jones Liquid and Glass. *Physical Review Letters* **60**, 2295-2298 (1988).

3 Mickel, W., Kapfer, S. C., Schröder-Turk, G. E. & Mecke, K. Shortcomings of the bond orientational order parameters for the analysis of disordered particulate matter. *The Journal of Chemical Physics* **138**, 044501 (2013).

4 Jungblut, S. & Dellago, C. Crystallization of a binary Lennard-Jones mixture. *The Journal of Chemical Physics* **134**, 104501 (2011).

5 Auer, S. & Frenkel, D. Numerical prediction of absolute crystallization rates in hard-sphere colloids. *The Journal of Chemical Physics* **120**, 3015-3029 (2004).

6 Filion, L., Hermes, M., Ni, R. & Dijkstra, M. Crystal nucleation of hard spheres using molecular dynamics, umbrella sampling, and forward flux sampling: A comparison of simulation techniques. *The Journal of Chemical Physics* **133**, 244115 (2010).

7 Sohn, S., Xie, Y., Jung, Y., Schroers, J. & Cha, J. J. Tailoring crystallization phases in metallic glass nanorods via nucleus starvation. *Nat. Commun.* **8**, 1980 (2017).

8 Uhlmann, D. A kinetic treatment of glass formation. *J Non Cryst Solids* **7**, 337-348 (1972).

9 Legg, B. A., Schroers, J. & Busch, R. Thermodynamics, kinetics, and crystallization of Pt57. 3Cu14. 6Ni5. 3P22. 8 bulk metallic glass. *Acta materialia* **55**, 1109-1116 (2007).
