# Supplementary material for: Supercluster-coupled crystal growth in metallic glass forming liquids
Source: Nat Commun. 2019 Feb 22;10:915. doi: 10.1038/s41467-019-08898-4 (PMC6385493; doi:10.1038/s41467-019-08898-4)
Supplement: Supplementary file 3 — Description of Additional Supplementary Files [file 41467_2019_8898_MOESM3_ESM.pdf]

Description of Additional Supplementary Files

File Name: Supplementary Movie 1
Description: In situ TEM movie of a 80 nm nanorod upon cooling from the liquid state (900o C) to the isothermal crystallization temperature (420o C).

File Name: Supplementary Movie 2
Description: In situ TEM movie of a 80 nm nanorod upon heating from glass (30o C) to the isothermal crystallization temperature (420o C).

File Name: Supplementary Movie 3
Description: In situ TEM atomic resolution movie of a 23 nm nanorod upon rapid heating from a glass (30o C) to the isothermal crystallization temperature (360o C).

File Name: Supplementary Movie 4
Description: In situ TEM atomic resolution movie of a 20 nm nanorod upon rapid heating from a glass (30o C) to the isothermal crystallization temperature (340o C).

File Name: Supplementary Movie 5
Description: In situ TEM atomic resolution movie of a 35 nm nanorod upon gradual heating from a glass (30o C) to the isothermal crystallization temperature (340o C).
